# Examining the effects of social media storytelling on Gen Z supporting cultural heritage and sustainability in the United Arab Emirates

**DOI:** 10.3389/fsoc.2025.1653182

**Published:** 2025-12-19

**Authors:** Riadh Jeljeli, Faycal Farhi, Merhan Mohsen, Mohamed Mallek, Samira Setoutah

**Affiliations:** 1 Communication Program, College of Arts, Humanities and Social Sciences, University of Kalba, Kalba, United Arab Emirates; 2 Communication Program, College of Arts, Humanities and Social Sciences, University of Al Dhaid, Al Dhaid, United Arab Emirates; 3 Department of Communication and Multimedia, University College of Bahrain, Manama, Bahrain; 4 Department of Communication, College of Arts, Humanities and Social Sciences, University of Khorfakkan, Sharjah, United Arab Emirates

**Keywords:** social media, storytelling, Gen Z, cultural preservation, structural equation modeling, family cohesion

## Abstract

This study aims to examine how digital storytelling on social media contributes to family resilience, the preservation of cultural heritage, and the long-term sustainability of cultural identity among Gen Z in the United Arab Emirates. Grounded in the Theory of Family Resilience, this study examines how social media engagement starts emotional bonds, facilitates cultural transmission, and reinforces identity within family structures. Using a quantitative approach, data is gathered from individuals aged 18–27, using structured surveys, who actively use social media. Results indicated a positive effect of social media storytelling on family cohesion, Cultural identity preservation, and cultural sustainability in the United Arab Emirates. The study supported all three hypotheses, indicating that social media storytelling positively affects family cohesion, cultural identity preservation, and cultural sustainability among Gen Z in the UAE. It is found that sharing family traditions and cultural stories on social media strengthens their relationship with family members and helps sustain cultural values across generations. This storytelling promotes better communication, understanding, and a shared sense of identity, promoting awareness and long-term sustainability of cultural practices. Thus, this study provides insights and implications on the role of Gen Z in sustainable cultural values and promoting social sustainability through digital practices, particularly within the rich sociocultural context of the United Arab Emirates. Study limitations and recommendations for future studies are further highlighted accordingly.

## 1 Background

The digital world has dramatically transformed due to social media platforms' emergence and rapid development over the past 20 years. What started as simple online networks has developed into complex systems driven by advanced algorithms, fundamentally changing how people connect, communicate, and interact with their surroundings (Kullolli and Trebicka, 2023). These changes have been fuelled by steady advancement in digital technology and communication tools, leading to higher-quality text, images, audio, and video content (Ali et al., 2025). A critical part of this digital transformation has been the impact of Generation Z, a group raised in an environment where digital connectivity is the norm. Their interaction with social media stands out not just because of how frequently or how long they use these platforms, but because of their unique ability to adapt to the constant stream of updates and innovations (Farhi et al., 2022). As a result, there has been a significant surge in younger users who engage with social media in highly instinctive and proficient ways. For example, recent data indicated that the United Arab Emirates had approximately 9.46 million internet users, reflecting a 99% internet penetration rate as of early 2024 (DataReportal, 2024). During the same period, social media users reached around 10.73 million, exceeding the total population and accounting for 112%. Also, the country had about 20.96 million active mobile connections, representing 21.9% of the population (DataReportal, 2024). This data also indicates approximately 2.58 million social media users aged 18 to 27 in the United Arab Emirates (DataReportal, 2024). This data shows that many social media users belong to Gen Z in the country. Despite the increasing prevalence of social media use among the young generation (Zheltukhina et al., 2019; Kullolli and Trebicka, 2023), there is limited empirical research on how social media storytelling influences the preservation of cultural heritage and family cohesion within the unique sociocultural context of the United Arab Emirates. This gap restricts a comprehensive understanding of the role that Gen Z specifically plays in sustaining cultural identity through digital platforms, underscoring the need for focused investigation into these dynamics. Notably, social media provides a pathway to digital storytelling, involves using digital tools, i.e., videos, images, audio, and social media platforms, to create and share personal or collective narratives (Lian et al., 2023). It combines traditional storytelling with modern technology, enabling individuals to express experiences, preserve cultural heritage, and engage audiences in interactive and multimedia formats (Lian et al., 2023).

However, this use has increased during and after the COVID-19 pandemic (Nicola and Ruspini, 2020; Savage et al., 2022), suggesting the importance of examining social media use in Gen Z's life as a crucial concern. Notably, this surge in social media use also indicated a strong interfamily connection caused by the COVID-19 pandemic. Due to strong social isolation measures, social media provided the only pathway to communicate with family members and friends to sustain social bonding and connection (Rajkumar and Sangeetha, 2021). Today, Ansie and Mbamba (2024) consider family communication to be one of the most significant aspects of social media for young users, providing venues to express affection and promote mutual understanding. Families are usually seen as the foundation of society, as the strength and unity within communities stem from the close-knit relationships formed within individual family units (Khalili et al., 2024). It is assumed that social media plays an important role in uniting family members, providing a space for young users to stay linked with others (Kacane and Hernández-Serrano, 2023). Notably, Generation Z (born between 1997 and 2012) represents a digitally native cohort known for their deep engagement with social media and preference for visual, interactive content (Youssef et al., 2024). In the UAE, Gen Z holds a unique cultural position, balancing traditional values with increasing global exposure. Social media storytelling, particularly through platforms like Instagram and TikTok, has become a modern tool for cultural transmission. In this context, storytelling allows young users to share family traditions, reinforce cultural identity, and stay connected across generations (Ansie and Mbamba, 2024). During globalization and societal change in the UAE, digital storytelling plays a crucial role in maintaining heritage and sustaining cultural values. This study explores how such practices contribute to family cohesion and long-term cultural sustainability among Gen Z in the UAE.

### 1.1 Study background

Digital storytelling is the use of digital multimedia tools, including text, images, video, audio, and interactive content, to narrate personal or collective experiences, usually in a narrative format (Dowling and Miller, 2019). It blends traditional storytelling methods with modern technology to create engaging, emotionally resonant content. For this study, social media storytelling is defined as the act of sharing personal, familial, or cultural narratives on social networking platforms such as Instagram, TikTok, YouTube, or Snapchat (Garber-Barron and Si, 2013). This includes visual posts, short videos, captions, hashtags, or live interactions that highlight family traditions, customs, values, and shared identities. It is a subset of digital storytelling specifically shaped by the interactive and public nature of social media platforms. In this context, social media storytelling is positioned as a contemporary form of cultural transmission and identity reinforcement among Gen Z in the UAE (Zubareva, 2020; Zakarneh and Annamalai, 2024). The United Arab Emirates (UAE) is a rapidly modernizing society with a diverse and predominantly expatriate population. Despite this, preserving Emirati cultural identity remains a national priority, supported by government programs that emphasize heritage conservation and the reinforcement of traditional values. For Generation Z, who steer both global digital cultures and local traditions, maintaining cultural continuity is especially important. Social media storytelling provides a modern avenue for young people to engage with, express, and sustain their cultural heritage amid globalization and societal change, helping to ensure the longevity of the UAE's distinctive cultural identity (Radwan, 2022).

### 1.2 Study aims, gaps, and significance

Considering an increased social media use during the past few years, it is critical to examine its effect on cultural and family heritage in the United Arab Emirates. An increased reliance on these social media platforms among Gen Z people depends on empirical insights regarding how they affect family communication, leading to a strong culture and sustainability in the country. Hence, this study aims to analyse the effects of social media storytelling on Gen Z in terms of supporting cultural heritage and sustainability in the United Arab Emirates. The special focus remains on storytelling as a prevalent practice among family members that reinforces cultural persistence among younger generations. This study is significant as it provides greater insight concerning Gen Z's social media use in the current era of digitalization. It also highlights the significance of digital technology in supporting family relations, leading to acknowledging one's cultural identity. Besides, it emphasizes the influence of social media as an important technology affecting social life and communication practices of younger generations in the United Arab Emirates.

## 2 Literature review

Social media storytelling has emerged as a powerful contemporary form of narrative expression, blending traditional storytelling with digital media formats to engage audiences emotionally and culturally (Ansie and Mbamba, 2024). Generation Z are digital native whose interaction with social media is not only frequent but also creative and identity-forming (Ibrahim Zakarneh et al., 2021). Preservation of cultural heritage, the collective memory, customs, and values that define communities, is increasingly viewed through the lens of sustainability, emphasizing the need to maintain these cultural practices amid rapid societal changes (Cueva et al., 2013). This literature review progressively examines these concepts, culminating in a focused analysis of the UAE, where unique demographic and cultural factors influence Gen Z's role in cultural sustainability through social media storytelling.

### 2.1 Family resilience theory

Resilience refers to the capacity to endure and recover from major life changes, emerging stronger and better equipped to cope. It is a dynamic process that supports positive adjustment and growth when faced with serious difficulties or hardships (Masykur and Adam, 2024). One of the main difficulties individuals and families face is sustaining or regaining healthy functioning when dealing with hardships or other transitions (Henry and Harrist, 2022). Drawing from concepts like individual resilience, family systems, and family stress models, many researchers and professionals now adopt the family resilience approach. This perspective highlights the capability of families to tap into their strengths and resources to continue functioning well, even when confronted with changes. Family resilience theory also supports the current study based on the transition from traditional media to new media, where communication and exposure to family remain comparatively easier. Also, after the COVID-19 pandemic, the digital transition and an ever-increasing number of young social media users reflected how they prefer digital platforms to communicate with their peers, friends, and family members. This communication is visible in the post-pandemic era as family members are linked (Ukaegbu et al., 2022). As noted by Liang et al. (2021), social media has opened up new opportunities for sharing family cultural heritage with a broader young audience. It is key in raising awareness about preserving family traditions and history. These platforms allow people to learn about their family norms, backgrounds, and what makes them unique. When individuals post stories and older members' experiences connected to their cultural roots, it helps build a stronger sense of belonging, collective identity, and resilience among family groups (He, 2022). A study by Shao et al. (2023) further explored how social media affects the promotion of cultural heritage among the young generation. Based on an analysis of 29 selected articles from two databases, the results showed that social media can broaden the reach of cultural heritage awareness and encourage users' interest and engagement among young users.

### 2.2 Social media storytelling and family cohesion

Digital storytelling is the use of digital multimedia tools, including text, images, video, audio, and interactive content, to narrate personal or collective experiences, usually in a narrative format (Dowling and Miller, 2019). It blends traditional storytelling methods with modern technology to create engaging, emotionally resonant content. For this study, social media storytelling is defined as the act of sharing personal, familial, or cultural narratives on social networking platforms such as Instagram, TikTok, YouTube, or Snapchat (Garber-Barron and Si, 2013). This includes visual posts, short videos, captions, hashtags, or live interactions that highlight family traditions, customs, values, and shared identities. It is a subset of digital storytelling specifically shaped by the interactive and public nature of social media platforms. In this context, social media storytelling is positioned as a contemporary form of cultural transmission and identity reinforcement among Gen Z in the UAE.

Similarly, Family cohesion is a key indicator of how well a family functions overall. Cohesion represents the emotional intimacy among family members and the level of autonomy each person experiences within the family structure (Lian et al., 2023). Researchers worldwide have shown increasing interest in investigating how social networking sites impact family relationships. However, many raised concerns over how social media use may affect individuals' behavior and shift their focus away from family and social connections. Several studies have highlighted prominent changes in how people interact within their families and social circles due to their engagement with social media platforms (Ali et al., 2024). However, existing literature (Meimaris, 2018; Bogdan et al., 2023) also witnessed social media as a source of family cohesion, through communication, sharing audio-video content, strengthening Cultural Heritage appreciation and awareness among young users. Tamme and Siibak (2012) further affirmed the role of social media, particularly storytelling from old users, in strengthening family bonding and cultural awareness in Estonia. Findings showed that new media significantly support and revive intergenerational communication and heritage transmission, with platforms like Skype, instant messaging, and Facebook fostering shared values and stronger family bonds, regardless of physical distance. Another study by LeBouef et al. (2023) also analyzed how college students use Snapchat to sustain and strengthen cultural awareness using a mixed-method approach. Based on interviews with 12 students, the study found that the app significantly supports family cohesion, raising awareness about family norms and heritage. Students described Snapchat as their primary mode of staying in touch with loved ones. The content analysis further emphasized three key ways Snapchat contributes to family cohesion: by helping to overcome physical distances, creating casual possibilities for interaction, and maintaining a sense of shared family identity. The results suggest that family cohesion and awareness habits are shifting as new digital platforms become widely adopted across generations (Jeljeli et al., 2024).

**H1**. Social media storytelling positively affects family cohesion among Gen Z in the UAE.

### 2.3 Social media storytelling and cultural identity preservation

The internet and social media have become critical tools for expressing and shaping cultural identity in today's digital age (Ladzekpo et al., 2024). These platforms present individuals and communities with new ways to explore, affirm, and share their cultural heritage. They help keep traditions alive while allowing for creativity and reinvention in a rapidly changing world (Wibowo et al., 2023). Online spaces bring together people with shared cultural interests, providing a sense of connection and support (Radwan, 2022). As digital environments evolve, they challenge old ideas of identity and introduce more synthesized and diverse expressions of culture. Through digital storytelling, people can document their lives, break stereotypes, and facilitate a deeper understanding of cultural diversity, emphasizing the importance of digital awareness and inclusive online spaces (Sádaba et al., 2020; Tovbych et al., 2020). Ajitoni (2024) empirically scrutinized how social media storytelling influences cultural identity and the role of social media in shaping contemporary cultural narratives. The results revealed that social media storytelling helps preserve and express cultural identities, promotes collective participation in narrative creation, and raises ethical concerns around representation and appropriation, indicating how individuals and communities reshape their cultural stories in digital spaces. Judijanto (2024) also examined how social media affects cultural identity formation by conducting a detailed literature review. Using a qualitative approach based on secondary data, the findings suggest that while social media allows broader cultural expression and strengthens identity, it raises concerns about cultural uniformity, loss of traditions, and the spread of stereotypes, emphasizing the need for responsible use and policy development to protect cultural diversity.

**H2**. Social media storytelling positively affects Gen Z's cultural identity preservation in the UAE.

### 2.4 Social media storytelling and support of cultural sustainability

Cultural heritage is crucial in sustaining social unity and passing human values through generations (Alivizatou, 2011). Digital technologies, particularly audiovisual tools, effectively document and preserve cultural expressions, customs, and traditions. Digital platforms provide engaging, multimedia experiences that help keep cultural stories alive (Lenzerini, 2011). Social media provides a useful space for efforts in preserving cultural heritage. It allows individuals to participate actively in heritage-related conversations and initiatives (Freeman, 2010). Online communities have flourished in the Web 2.0 era, creating opportunities for people to connect and collaborate without geographical limitations, as they are usually centered around specific cultural practices or heritage sites (Psomadaki et al., 2019). These platforms promote broader engagement and cross-cultural understanding. Users share personal memories, cultural experiences, and emotional ties, contributing to a collective sense of place and identity (Díaz-Andreu, 2017). A study by Liang et al. (2021) examined how social media contributes to digital community engagement in cultural heritage management through a systematic literature review. Reviewing 248 relevant articles and selecting 19 for detailed analysis indicated that social media is important in facilitating public participation, enabling broader community involvement, and supporting inclusive decision-making in heritage conservation, particularly in rapidly urbanizing areas. However, the fast pace of modern life, globalization, and rapid technological change threaten cultural diversity. In this context, using innovative digital methods to present heritage in ways that appeal to younger audiences is becoming increasingly critical to keep cultural identities relevant and resilient (Cahyani et al., 2023; Funk, 2024). Given the cited literature and theoretical debate, this research proposed the following hypotheses.

**H3**. Social media storytelling positively affects Gen Z's support of cultural sustainability in the UAE.

## 3 Methodological approaches

### 3.1 Research design and data analysis

This study involves a quantitative approach, using structured surveys as the preliminary tool for data collection, as suggested by (Bayley 2013). The purpose of this approach was to generate precise and generalizable findings. After data collection, the responses were carefully reviewed to identify and correct any inconsistencies. Once the data was obtained and finalized, the dataset was coded and entered into SPSS for analysis, while SmartPLS was used to perform Structural Equation Modeling (SEM).

### 3.2 Sampling approaches

For this study, Generation Z is defined as individuals born between 1997 and 2012, consistent with widely accepted generational boundaries (Zakarneh and Annamalai, 2024). This definition ensures clear differentiation from preceding generations, such as Millennials, and facilitates precise sample selection targeting social media users aged 18–27 within the UAE. This study adopts a stratified sampling approach grounded in the country's unique demographic background to ensure representative insights into Gen Z's perspectives. Recognizing the geographical and cultural diversity across the Emirates, the sample is proportionally stratified based on the estimated population distributions, ensuring that voices from highly urbanized and less-populated regions are equitably highlighted. The sample size is determined using Yamane's sample calculation formula. As mentioned earlier, currently there are 2.58 million Gen Z social media users in the UAE (DataReportal, 2024), a sample size of 400 respondents at a ∓ 5% margin of error, is found statistically suitable to draw a reliable conclusion (Adam, 2020). Notably, of 400 respondents, 35% are drawn from Dubai, 30% from Abu Dhabi, 15% from Sharjah, and the remaining 15% are distributed across Ajman, Ras Al Khaimah, Fujairah, and Umm Al Quwain. Once the data was gathered, the acquired responses were carefully evaluated and counted. It was found that 53 responses were missing, finalizing 347 responses for further data analysis. This total number of responses indicates a response rate of 86.5%, which remained higher than the minimum threshold of 60% Deutskens et al., (2004). Table 1 summarizes the respondents' demographics.

**Table 1:** Details of study questionnaire.

**Variables**	**Sources**	**Items**
Social media storytelling	Malkawi et al., 2019; Nicoli et al., 2021	How often do you share personal stories on social media? How often do you use social media to tell stories about your family? I enjoy sharing personal stories about my family on social media. Social media allows me to express my personal experiences. Social media allows my family members to share their celebrations. I believe that social media storytelling can help preserve important family memories.
Family cohesion	Ukaegbu et al., 2022; LeBouef et al., 2023	I use social media to share family traditions, memories, or stories with my family members. Social media storytelling helps me feel more connected with my family, even if they live far away. Maintaining my family's cultural identity through stories shared on social media is important to me. Social Media storytelling has improved communication and understanding among my family members. Social media storytelling has improved understanding among my family members.
Cultural identity preservation	Bucholtz and Hall, 2005; Tovbych et al., 2020; Ajitoni, 2024	Sharing stories about my culture on social media helps preserve my cultural identity. I believe social media storytelling contributes to maintaining cultural traditions. Social media allows users to express cultural values to a wider audience. Social media storytelling positively impacts preserving cultural identity for future generations. I feel more connected to my cultural heritage by sharing stories on social media.
Cultural sustainability	Stuedahl and Mörtberg, 2012; Chen and Chen, 2025	Social media storytelling helps promote the long-term sustainability of cultural practices. Sharing stories on social media is important in preserving family traditions for future generations. Social media platforms contribute to the sustainability of my family traditions. Cultural sustainability is supported by raising awareness of cultural practices through social media storytelling. Social media storytelling helps maintain the relevance of family traditions in modern society.

### 3.3 Data collection instrument

A closed-ended questionnaire was developed using a five-point Likert scale, with survey items adapted from established research consistent with the current study's objectives. Table 2 provides a detailed breakdown of the questionnaire items and their sources. The reliability and validity of the survey instrument are evaluated in the next steps using Confirmatory Factor Analysis (CFA) to test convergent validity, construct reliability, and discriminant validity.

**Table 2:** Summary of respondents' demographics in the current study.

**Variables**	**Constructs**	***N***	**%**
Gender	Male	166	47.8%
	Female	181	52.2%
Age group	18–20 years	53	15.3%
	21–23 years	119	34.3%
	23–25 years	161	46.4%
	26–27 years	14	4.0%
Education	Undergraduate	143	41.2%
	Graduate	129	37.2%
	Masters	59	17.0%
	Doctorate	16	4.6%
Emirates	Dubai	138	39.7%
	Abu Dhabi	112	32.7%
	Sharjah	51	14.6%
	Others (Ajman, Ras Al Khaimah, Fujairah, and Umm Al Quwain)	46	13.2%

## 4 Analysis and findings

**Figure 1:**
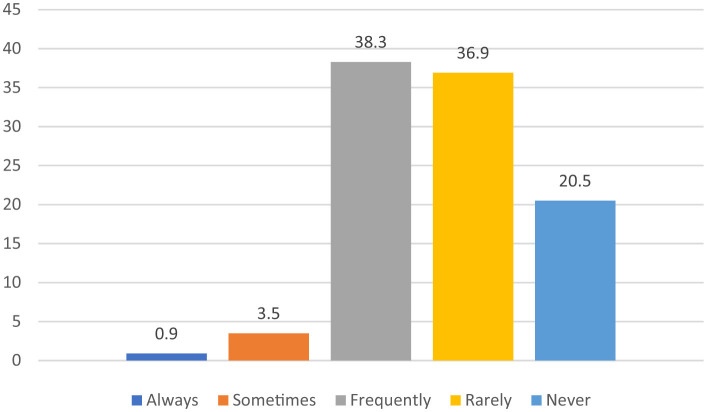
Percentage of sharing personal stories on social media.

This section comprises data analysis and reporting on results. First, descriptive results are reported, including responses regarding social media use for sharing stories concerning family and personal life. As shown in the Figure 1, shows the percentages of responses regarding sharing personal stories on social media. According to 38.3% of respondents, they frequently share personal stories on social media, 36.9% rarely share, while 20.5% rarely share their personal stories online. However, 3.5% marked “sometimes”, and only 0.9% revealed that they always share personal stories on social media.

**Figure 2:**
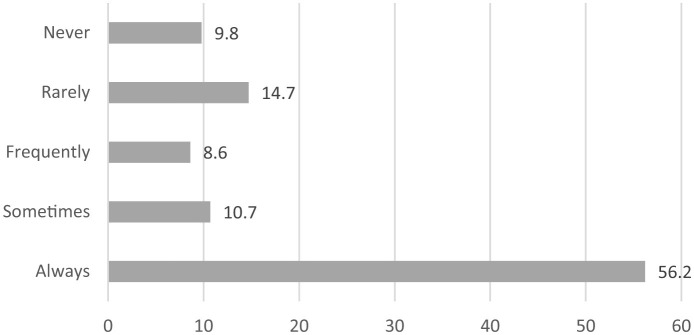
Percentage of social media use for storytelling about family.

Figure 2 further shows the percentage of social media use for storytelling about family. As visible, 56.2% revealed that they always use social media for storytelling about family, 14.7% rarely use it, and 10.7% prefer sometimes using it. Besides, 9.8% marked “never,” and 8.6% indicated that they frequently use social media for storytelling about family.

### 4.1 Structural equation modeling

The next step involves structural equation modeling (SEM) to test the reliability and validity of the measurement tool, and further, assessing the proposed hypotheses (Farhi et al., 2021; Hidayat and Wulandari, 2022). First, the results of Confirmatory Factor Analysis (CFA) revealed that most of the loading values, related to each item, surpass the threshold of 0.5. Meanwhile, the average variance extracted values (AVE) also exceed the threshold of 0.5 (Social Media Storytelling 0.592, Family Cohesion 0.515, Cultural Identity Preservation 0.602, and Cultural Sustainability 0.589).

Further, assessing the construct reliability revealed that Cronbach Alpha (CA) (Social Media Storytelling 0.740, Family Cohesion 0.734, Cultural Identity Preservation 0.715, and Cultural Sustainability 0.743) values and Composite Reliability (CR) values (Social Media Storytelling 0.827, Family Cohesion 0.757, Cultural Identity Preservation 0.743, and Cultural Sustainability 0.789) also surpass the threshold 0.7 (Amirrudin and Nasution, 2021). Overall, these results indicate that internal consistency exists among the study variables. Table 3 represents the results of confirmatory factor analysis.

**Table 3:** Confirmatory Factor Analysis (CFA).

**Variables**	**Questionnaire statements**	**Loads**	**AVE**	**CA**	**CR**
Social media storytelling	SMT1	0.514	0.592	0.740	0.827
	SMT2	0.843			
	SMT3	0.936			
	SMT4	0.947			
	SMT5	0.921			
	SMT6	0.533			
Family cohesion	FCO1	0.939	0.515	0.734	0.757
	FCO2	0.596			
	FCO3	0.226			
	FCO4	0.386			
	FCO5	0.668			
Cultural identity preservation	IDE1	0.801	0.602	0.715	0.743
	IDE2	0.276			
	IDE3	0.840			
	IDE4	0.757			
	IDE5	0.799			
Cultural sustainability	SUS1	0.464	0.589	0.743	0.789
	SUS2	0.843			
	SUS3	0.936			
	SUS4	0.947			
	SUS5	0.924			

**Figure 3:**
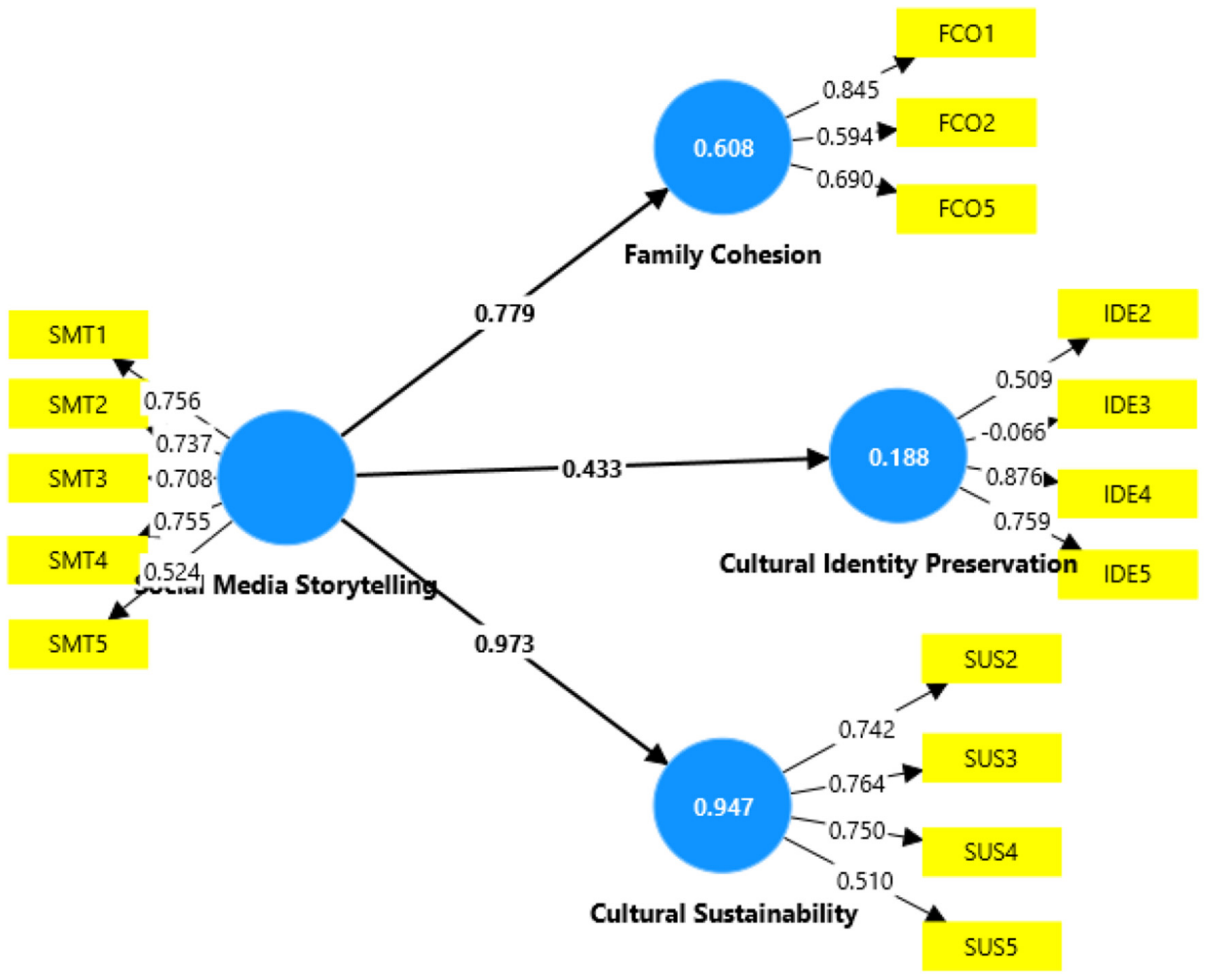
Final structural model.

As some items indicate lower loading values (FCO3, FCO4, IDE1, SUS1), they are removed to test the goodness of fit. The relevant test, including the structural model testing, is applied to determine how well the obtained values fit the expected values (Mérigot et al., 2010). Figure 3 shows the model finalized for the structural model testing after removing the items with lower loading values. Assessing the goodness of fit indicates Standardized Root Mean Square (SRMR) value at 0.0035, Non-fit Index value 0.822 (b/w 0-1), Tucker and Lewis (TLI) value 0.950 (b/w 0–1), and Chi-square value at 1.53574 (< 3.0). Overall, these results indicate a good fit for the model.

Divergent validity of measurement tool is further assessed using Fornell-Larker criterion suggested by Cheung and Wang (2017). The relevant assessment involves computing the squares of each AVE value and comparing them with the correlations mentioned in Table 4. Thus, it is found that the squares of all the AVE values are not only greater but also do not correlate with the other relevant values in the table. This indicates that divergent validity exists in the measurement tool.

**Table 4:** Divergent validity testing.

**Divergent validity testing**	**Cultural identity preservation**	**Cultural sustainability**	**Family cohesion**	**Social media storytelling**
Cultural identity preservation	**0.636**			
Cultural sustainability	0.460	**0.699**		
Family cohesion	0.545	0.650	**0.722**	
Social media storytelling	0.464	0.661	0.702	**0.651**

Square roots of AVE values.

Before testing the study hypotheses, the predictive power and effect size of Social Media Storytelling as an independent variable are tested. First, testing the R^2^ revealed a strong predictive power of Social Media Storytelling on Cultural Sustainability, explaining 94.7% of variance, and a moderate predictive power on Cultural Identity Preservation, with 60.8% of variance. However, the effect size on Cultural Identity Preservation remains weak, explaining 18.8% of variance. Concerning the effect size, social media storytelling greatly affects cultural sustainability (17.943) and family cohesion (1.548). The effect size on Cultural Identity Preservation remains moderate (231), which is considered acceptable based on Cohen's criterion for f^2^ determination (Selya et al., 2012). Table 5 summarizes the results of R2 and f2 analyses.

**Table 5:** R^2^ and f^2^ analysis.

**Variables**	***R^2^***	***f^2^***
Family cohesion	0.608	1.548
Cultural identity preservation	0.188	0.231
Cultural sustainability	0.947	17.943

**Figure 4:**
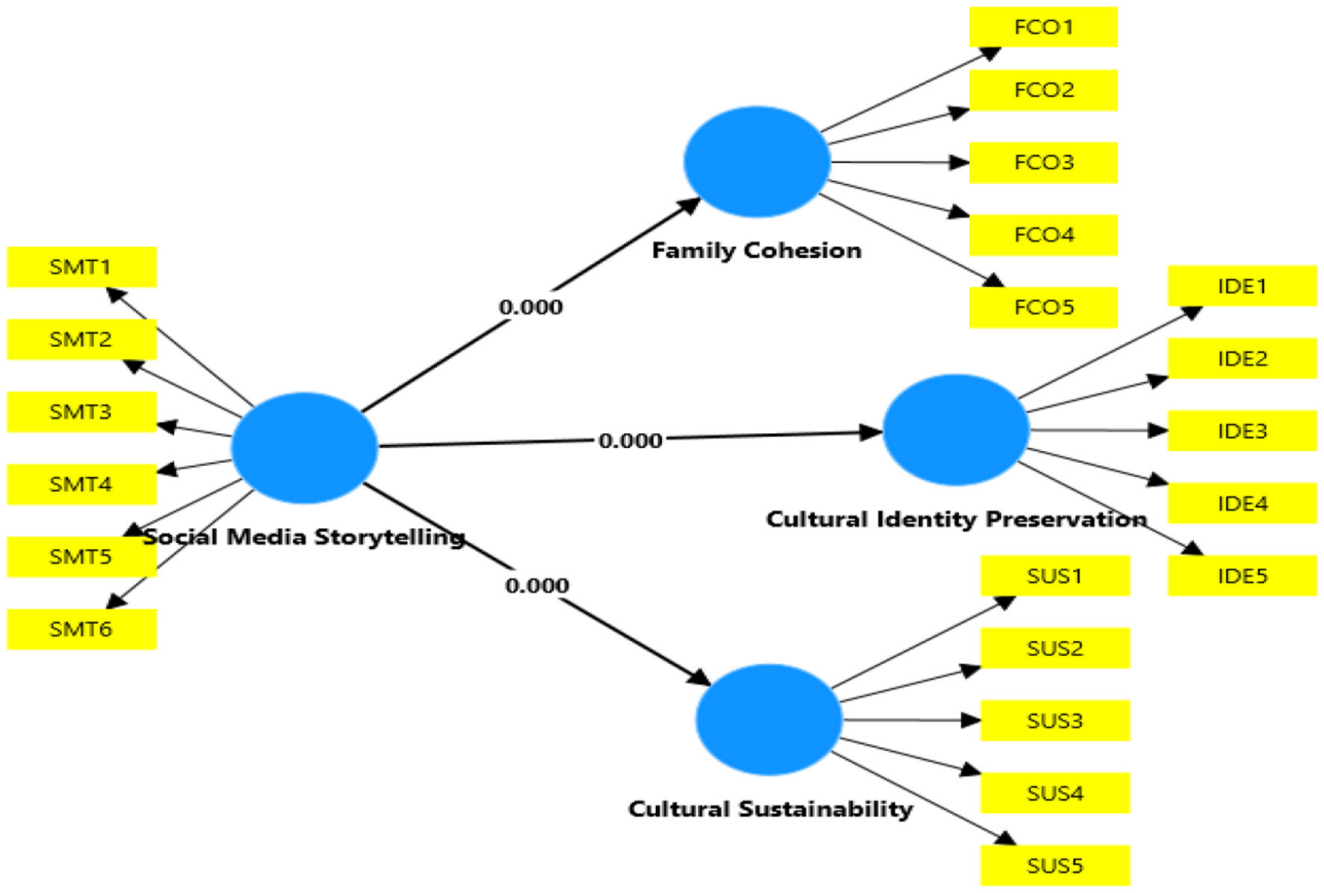
Final structural model (hypotheses testing).

Finally, hypotheses are tested using path analysis that involves beta-coefficient values (β), t-statistics, and path values. Table 6 represents the results of path analysis in the Table 5. First, the first hypothesis is assessed, indicating a positive effect of social media storytelling on family cohesion among Gen Z in the UAE. Results show the beta coefficient value (β) of 0.779, and t-statistic of 24.637, along with the significance value of 0.000 (*p* < 0.05), supporting the first study hypothesis. The second hypothesis regarding the positive effects of social media storytelling on cultural identity preservation also remains significant. With the beta coefficient value (β) of 0.443 and a t-statistic of 9.305, along with the significance value of 0.000 (*p* < 0.05), the analysis indicated a strong positive effect of social media storytelling on cultural identity preservation. Finally, the proposed effect of social media storytelling on cultural sustainability is tested. With the beta coefficient value of coefficient value (β) 0.973, t-statistic 27.398, and the significance value of 0.000 (*p* < 0.05), the third hypothesis is also supported. Regarding the paths, the path between social media storytelling and cultural sustainability remained strongest (0.973). Followed by a path between media storytelling and family cohesion (779), the path between media storytelling and cultural identity preservation remained the weakest (0.433). Figure 4 shows the results of path analysis, including *p*-values.

**Table 6:** Hypotheses testing.

**Hypotheses testing**	**Mean**	**SD**	**β**	**t-statistics**	**Path**	**Decision**
Social media storytelling ➔ family cohesion	1.174	0.048	0.779	24.637	0.000	Supported
Social media storytelling ➔ cultural identity preservation	0.635	0.068	0.433	9.305	0.000	Supported
Social media storytelling ➔ cultural sustainability	1.345	0.049	0.973	27.398	0.000	Supported

## 5 Discussion

Storytelling has long played a crucial role in human society, acting as a powerful entertainment mechanism, learning, communication, and preserving cultural traditions (Díaz-Andreu, 2017). It is deemed an important and distinctive part of the human experience, enjoyed by people of all ages and backgrounds. No matter the form, whether it is a poem, drama, novel, myth, or fairy tale, stories captivate audiences. While storytelling once depended on oral traditions and written texts, modern technology has extended its reach through print, radio, television, film, and digital platforms (Judijanto, 2024). Kalantari et al. (2023) further argued that stories have long functioned to maintain cultural values and heritage. People usually use storytelling to pass down historical knowledge to younger generations, with family histories commonly shared through personal accounts, diaries, or written narratives. Regular storytellers tend to share stories that mirror their opinions and support their families, and themselves, to stay connected and communicate with their loved ones (Liaqat et al., 2023).

Regarding the current findings, study respondents agreed that they often share personal and family stories on social media. According to the respondents, they enjoy sharing personal stories about family on social media as it allows them to express their personal experiences. It is also found that social media allows study respondents' family members to share their celebrations online, as these platforms can help preserve important family memories. In line with the argumentation by Alivizatou (2011). As noted, the broader usage of social media and other online platforms that support user-generated content has led to an increased use of digital storytelling. This storytelling involves personal narratives and self-expressive content created by everyday individuals and shared online. Accordingly, Prins (2016) argued that people now tell their stories through blogs, social media posts, short autobiographical videos, and online profiles. Unlike conventional storytelling, digital storytelling includes interactive elements, allowing audiences to consume content and respond, contribute, and shape the narrative. Both the storyteller and the audience become active participants, creating, interpreting, and reflecting together in this exchange (Pasha et al., 2022; Ali et al., 2025).

The study found that Generation Z in the UAE actively uses social media platforms like Instagram and TikTok to share personal and family stories that celebrate Emirati cultural heritage. For example, respondents reported posting about family gatherings during National Day or Eid celebrations, showcasing traditional clothing, i.e., the kandura and abaya, sharing local music, poetry, and proverbs unique to Emirati culture. These posts not only allow them to express their personal experiences but also serve as digital archives preserving important cultural memories. Importantly, social media facilitates interaction, friends and family comment, share, and contribute, transforming storytelling into a collective, dynamic practice. This engagement helps strengthen family cohesion and fosters a shared cultural identity, ensuring the transmission and sustainability of Emirati traditions amid rapid modernization and globalization.

Notably, cultural identity preservation manifests through different tangible and intangible practices that social media storytelling helps to sustain in the UAE. Examples include the traditional Emirati dress, such as the kandura for men and abaya for women, which symbolize national pride and heritage (Hills, 2013). Language use, especially the preservation of Arabic and local dialects, is also vital, as storytelling often includes phrases, proverbs, and poetry distinctive to Emirati culture. Oral storytelling remains an important practice within families, where elders share historical narratives, Bedouin tales, and religious teachings that reinforce communal values. Moreover, national celebrations such as National Day, Eid, and the annual Al Dhafra Festival are widely shared and documented on social media, allowing Gen Z to participate virtually, honor traditions, and promote cultural continuity. These examples illustrate how social media storytelling serves as a dynamic platform that not only preserves but also revitalizes Emirati cultural heritage amid modernization and globalization (Hopkyns, 2017).

Regarding the first study hypothesis, “**H1. Social media storytelling positively affects family cohesion among Gen Z in the UAE”**, the results remained supportive. A general agreement was found, as respondents indicated that they use social media to share family traditions, memories, or stories with family members. According to the respondents, social media storytelling helps them feel more connected with family, even if they live far away. They also agreed that maintaining their family's cultural identity through stories shared on social media is important, as it has improved communication and understanding among family members. Besides, they agreed that social media storytelling has improved understanding among family members. As noted by Ali et al. (2024), existing studies have also shown an increased relationship between social networking sites and family relationships. Notably, the significance of family cohesion lies in its power to promote a sense of identity and continuity (Milano et al., 2023). They are crucial for social structure, where traditions, values, and norms are transmitted across generations, building a harmonious sense of belonging (Cueva et al., 2013). This shared identity is important for individuals to link with their origins and communities to preserve harmony and strengthen social ties. Particularly, family cohesion and cultural sustainability play a key role in helping to understand the background of young people, which can greatly shape their perspectives on family dynamics and the community (Gulnora, 2024). Based on family resilience theory, the current study results also indicated how the young generation in the UAE focuses on social media storytelling to stay connected, leading to stronger effects on their family cohesion, cultural identity, and cultural sustainability. Despite a plethora of studies (Kolhar et al., 2021; Smith et al., 2021; Ghai et al., 2022) indicating a negative effect of social media use among Gen Z having negative effects on their family life and mental health, current study stands out a distinct evidence, suggesting the positive side of social media as a source of storytelling and resilient communication. Current study findings indicated that Gen Z in the UAE acknowledges the positive effects of social media as a useful tool today.

The second hypothesis, “**H2. Social media storytelling positively affects Gen Z's cultural identity preservation in the UAE”. Also remains accepted**. This implies that study respondents agreed that sharing stories about culture on social media helps preserve cultural identity, contributing significantly to maintaining cultural traditions. Respondent further indicated that social media allows users to express cultural values to a wider audience, positively preserving cultural identity for future generations. Respondents also agreed they feel more connected to cultural heritage by sharing stories on social media. These findings indicated a strong consistency with the existing literature (Radwan, 2022; Solomon et al., 2022). For example, Ladzekpo et al. (2024) examined the effect of storytelling and narrative on cultural identity and understanding social values through literature. Using secondary data to explore how literary narratives had contributed to shaping and expressing cultural identity indicated that storytelling played a crucial role in preserving cultural heritage, supporting societal norms, and facilitating a shared sense of identity.

Finally, the third hypothesis also supported proposing a positive effect of ”**social media storytelling among Gen Z's support of cultural sustainability in the UAE”**. The respondents strongly agreed that social media storytelling helps promote the long-term sustainability of cultural practices. Besides, sharing stories on social media is important in preserving family heritage for future generations, as they significantly contribute to promoting family traditions. Also, they agreed that cultural sustainability is supported by raising awareness of cultural practices through social media storytelling, as it helps maintain the relevance of family traditions in modern society. Botangen et al. (2017) also found the similar effect of social media in promoting cultural awareness and sustainability. The focus remained on how social media supports preserving and promoting indigenous knowledge among Igorot communities living abroad. Analysis of 20 Facebook posts and surveying 56 Igorot migrants, the results highlighted that social media plays a crucial role in facilitating cultural exchange, renewal, learning, and the persistent practice of indigenous traditions. Liang et al. (2021) also reviewed existing studies witnessing the effect of social media on cultural sustainability, indicating social media platforms significantly contribute to sustainable development by enabling broader public participation in cultural heritage management. It emphasized how these tools allow various stakeholders to engage in decision-making processes, encouraging inclusive and collaborative approaches to heritage preservation.

## 6 Conclusion and implications

This study highlights the significant role of social media storytelling in promoting stronger family connections and preserving cultural heritage across generations. The key findings indicate that families actively engage in sharing stories, traditions, and values through social media platforms, which help maintain cultural continuity and intergenerational solidarity. These digital narrations offer an accessible, strong, and interactive way for family members to connect, learn, and pass down collective memories in a format that resonates with younger generations. It is found that social media storytelling is particularly important in sustaining culture as it enables families to adapt traditions within contemporary contexts, making them relevant and engaging. It also provides a public yet intimate space for expression, identity, and values, contributing to a shared cultural understanding that surpasses physical boundaries. Especially for Gen Z, social media is more than just entertainment; it is a crucial platform for self-expression, community building, and cultural engagement. Recognizing its influence and potential, policymakers, educators, and families should consider social media as a critical tool to support cultural education and family dialogue. Embedding social media storytelling ensures that culture remains crucial, inclusive, and evolving in the digital age.

The results of this study indicate that social media storytelling holds powerful potential for reinforcing family relationships and preserving cultural heritage among Gen Z in the United Arab Emirates (UAE). When young people use social media platforms to share stories about their families, traditions, and personal experiences, it helps them feel more emotionally connected to their loved ones, especially in a fast-paced, digital world where face-to-face interactions may be limited. This continuous connection supports stronger family cohesion, showing that storytelling is more than a form of expression. It is a bridge that keeps family members emotionally close across distances. This study also implies that storytelling helps young individuals value and express their cultural identity. Gen Z gains a deeper sense of belonging and pride in their cultural background by posting and engaging with content related to traditions and heritage. Most significantly, these storytelling practices help ensure the long-term sustainability of cultural values and customs. In a rapidly evolving society, such practices link the past, present, and future. These insights highlight the need for educational institutions, community leaders, and policymakers to promote digital storytelling initiatives, as they can serve as powerful tools to strengthen international ties, promote cultural awareness, and support resilient, culturally rooted communities in the digital age.

This study also implies the importance of digital space as a modern cultural hub where traditions are not remembered but actively reinterpreted and kept alive. As Gen Z is adopting a digital lifestyle, their engagement with cultural storytelling on social media reflects a shift in how heritage is experienced and shared. Instead of relying only on traditional, face-to-face methods of passing down values, young people blend personal expression with cultural narratives in ways that resonate with their peers. This opens up new cultural education and engagement possibilities, making it more accessible, interactive, and relevant. The integration of storytelling into daily digital habits means that culture is not on the sidelines but embedded within the everyday experiences of younger generations. This evolving nature can help build culturally confident individuals who are better prepared to preserve and celebrate their heritage, while also embracing the tools of a globalized, digital world.

### 6.1 Limitations

This study fills important gaps in the existing literature but has some limitations that cannot be overlooked. First, this study is focused on only Gen Z in the United Arab Emirates (UAE), which indicates diversity in the sample. Future studies can involve a diverse sample to overcome this limitation. The second limitation includes using a single quantitative method for data gathering. The relevant limitation can be countered using additional approaches, i.e., mixed-methods, to delimit this scope. Finally, the third limitation includes geographical generalizability, as this study is conducted in the United Arab Emirates, highlighting the effect of social media storytelling. Future investigations can replicate the current study and conduct more investigations in different regions to acquire more in-depth insights.

## Data Availability

The original contributions presented in the study are included in the article/supplementary material, further inquiries can be directed to the corresponding author.
